# Recent Advancements in Enzyme-Based Lateral Flow Immunoassays

**DOI:** 10.3390/s21103358

**Published:** 2021-05-12

**Authors:** Donato Calabria, Maria Maddalena Calabretta, Martina Zangheri, Elisa Marchegiani, Ilaria Trozzi, Massimo Guardigli, Elisa Michelini, Fabio Di Nardo, Laura Anfossi, Claudio Baggiani, Mara Mirasoli

**Affiliations:** 1 Department of Chemistry “Giacomo Ciamician”, Alma Mater Studiorum-University of Bologna, Via Selmi 2, 40126 Bologna, Italy; donato.calabria2@unibo.it (D.C.); maria.calabretta2@unibo.it (M.M.C.); martina.zangheri2@unibo.it (M.Z.); elisa.marchegiani2@unibo.it (E.M.); ilaria.trozzi2@unibo.it (I.T.); massimo.guardigli@unibo.it (M.G.); elisa.michelini8@unibo.it (E.M.); 2 Department of Chemistry, University of Turin, Via P. Giuria 5, 10125 Turin, Italy; fabio.dinardo@unito.it (F.D.N.); laura.anfossi@unito.it (L.A.); claudio.baggiani@unito.it (C.B.)

**Keywords:** chemiluminescence, colorimetric, enzyme, lateral flow immunoassay, metal nanoparticles, nanozyme, point-of-care

## Abstract

Paper-based lateral-flow immunoassays (LFIAs) have achieved considerable commercial success and their impact in diagnostics is continuously growing. LFIA results are often obtained by visualizing by the naked eye color changes in given areas, providing a qualitative information about the presence/absence of the target analyte in the sample. However, this platform has the potential to provide ultrasensitive quantitative analysis for several applications. Indeed, LFIA is based on well-established immunological techniques, which have known in the last year great advances due to the combination of highly sensitive tracers, innovative signal amplification strategies and last-generation instrumental detectors. All these available progresses can be applied also to the LFIA platform by adapting them to a portable and miniaturized format. This possibility opens countless strategies for definitively turning the LFIA technique into an ultrasensitive quantitative method. Among the different proposals for achieving this goal, the use of enzyme-based immunoassay is very well known and widespread for routine analysis and it can represent a valid approach for improving LFIA performances. Several examples have been recently reported in literature exploiting enzymes properties and features for obtaining significative advances in this field. In this review, we aim to provide a critical overview of the recent progresses in highly sensitive LFIA detection technologies, involving the exploitation of enzyme-based amplification strategies. The features and applications of the technologies, along with future developments and challenges, are also discussed.

## 1. Introduction

Since their invention in the 1980s [1] and the launch on the market in 1984 with the pregnancy test by Unipath, a Unilever spin out at Colworth (UK), Lateral Flow Immunoassys (LFIAs) have become very popular for low-cost, easy-to-use, rapid testing in point-of-care (POC) applications [2,3]. Undoubtedly, LFIAs owe their success to the combination of the portability, rapidity and simplicity afforded by the strip format with the high specificity and sensitivity of immunological methods, which provides target analyte detection even in complex biological matrices without a tedious sample pretreatment. Moreover, the immunoassay takes place on a nitrocellulose membrane, in which sample and reagents spontaneously flow by capillarity, making this platform very attractive for its low cost and simplicity. LFIA-based devices have been developed for a wide range of analytes of clinical [4,5], veterinary [6,7], agrifood [8,9] and environmental [10,11] interest, enabling detection of proteins, nucleic acids, drugs, hormones, toxins, viruses and bacteria.

LFIAs offer several advantages, such as rapidity, simplicity, easy results interpretation, in-field applicability, prolonged shelf-life, small volumes required, relatively low-cost production, good sensitivity and specificity and possibility of integration with electronics and electrochemical systems. Nevertheless, the technology still has some limitations, which attracted great interest in the research world and need to be overcome to allow a further expansion of applications of LFIA. Long-standing criticisms concern limited sensitivity and reproducibility, scarce quantification capability, challenging implementation of multistep assays and integration with electronic/electrochemical systems, and limitations in multiplexing ability due to both possible cross-reactivity issues and the limited space available on the nitrocellulose strip placing separate target analyte capture areas.

LFIAs were originally conceived as rapid qualitative tests, suitable for detecting the presence/absence of a target analyte in the sample through a visual colorimetric approach. Indeed, specific bio-reagents are immobilized in defined areas of the strip, where the formation of colored bands due to the accumulation of suitably labeled species yields a yes/no information. In particular, the analytical response is observed in the so-called test line (T-line), while a second control line (C-line) allows to verify that the test was correctly performed and therefore that results were reliable. Use of various labels and coupling with sensitive, portable and easy-to-use detectors have been extensively explored to develop ultrasensitive quantitative LFIAs, leading to fruitful achievements and remarkable improvements [12].

Indeed, several modifications of the standard LFIA design and/or structure have been reported, regarding both the materials used as transducers for the signal generation [13] and the methodologies employed to mediate the LFIA response or to improve the performance of the device with different enhancement strategies [14].

Metal nanoparticles (MNPs)-based labels, especially gold NPs (AuNPs) and silver NPs (AgNPs), have been widely applied in LFIA, as they combine good analytical performance, high sensitivity, and adaptability to several detection formats. Indeed, MNPs labels can provide quantitative information through spectroscopy or optical imaging of the LFIA membrane, using very simple instrumental setup, generally comprising a light source, optic lenses for optimal light signal collection and easy-to-use detectors (e.g., complementary metal oxide semiconductor (CMOS) or charge-coupled device (CCD) cameras). By measuring the signals in correspondence of the T- and C-lines and signal correction with respect to background signals, the concentration of the target analyte can be obtained by interpolation on a pre-stored calibration curve [15]. In this context, numerous optical LFIA devices were commercially proposed, along with their dedicated portable readers, for different applications, such as medical diagnostics [16], food safety [17], environmental monitoring [18], and veterinary diagnostics [19]. The recent trend of smartphone-based colorimetric LFIAs has attracted great attention, since it exploits smartphones’ high-performance CMOS-cameras equipped with flashlight sources and data processing capabilities [2,3,15,20,21,22,23].

Owing to their great versatility, AuNPs are also suitable as labels for Surface Enhanced Raman Scattering (SERS)-based methods, which provide ultrasensitive LFIA systems [24,25] by exploiting high-performance spectrometers. The high sensitivity offered by SERS-based LFIA method, however, is counterbalanced by the complexity and high costs of the required instrumentation for detection (e.g., high-power Raman laser and spectrometer). Technological advances are necessary to make this technology low-cost and easy-to-use to promote its application for onsite use [15].

MNPs have also been proposed as labels for thermal-based detection, exploiting their high light absorption capacity in specific spectral ranges, which is called Localized Plasmon Surface Resonance (LSPR). This phenomenon results in heat production when MNPs are illuminated by a light whose wavelength matches with the LSPR absorption band. The temperature increase is proportional to the amount of tracing particles, and therefore measurement of this photo-thermal (PT) response provides quantitative analyte detection [26,27,28].

Magnetic-NPs have also been proposed for LFIA applications. Indeed, by applying an external magnetic field, a stray field is generated from the magnetic-NPs and it can be detected exploiting different detection approaches, including giant magnetoresistance [29,30], tunneling magnetoresistance and magnetic particle quantification [31]. LFIAs based on the magnetic response of magnetic-NPs offer several attractive features over other sensing modalities [32,33]. Indeed, they show very low detection limits and no background signals, since biological specimens (e.g., urine, serum and blood) are free from magnetic interferents. These LFIAs can also be readily configured to perform multiplexed detection [31,34]. Nonetheless, the equipment required for the magnetic measurements is generally bulky, lab-based and expensive. Some miniaturized readout systems have been recently described, providing the possibility of transforming these sensors into POC devices [35,36].

Highly sensitive LFIA can also be performed using fluorescent (FL) labels (e.g., dyes, quantum dots, and fluorescent nanoparticles). With respect to colorimetric detection, FL-based detection offers high sensitivity and low background, enabling quantitative measurement of trace analytes, such as DNA or tumor markers, with high signal-to-noise ratio [37,38]. As in the case of SERS- and magnetic-based LFIAs, the instrumentation for FL measurements is more complicated and expensive than that for the colorimetric approach. Indeed, high-power light sources, such as lasers or laser diodes, are necessary for the efficient excitation of fluorophores, as well as dichroic mirrors or emission filters are required to selectively measure FL emission. Smartphones can be used as alternative low-cost platforms for FL-based LFIAs, although they must be equipped with additional optical accessories [39,40,41].

As an alternative, electrochemical (EC) detection has also been proposed in combination with LFIA technique. This approach is based on the use of redox tracers which can be quantitatively measured exploiting electron transfer processes that happen at the interface of dedicated electrodes on the LFIA membrane. EC-LFIA systems have been reported in literature based on amperometric, voltammetric and impedimetric detection methods [42,43]. In addition, integration of electrodes on the LFIA strip enabled the use of electrochemiluminescent (ECL) tracers for ECL-LFIA applications, providing high sensitivity, wide detection range and high reproducibility [44]. However, EC-LFIA systems still suffer from limited applicability, as they present several disadvantages in terms of design of the LFIA system, relatively high costs and complexity of procedures, which involve the addition of specific reagents and require to perform the assay in the solution phase. Further technological advancements are thus needed to ensure greater diffusion of these systems [15].

Enzymes have been proposed as labels for LFIAs to provide signal amplification, thus enhancing assay performance for various transduction principles, and greater versatility. Indeed, enzyme-linked immunosorbent assays (ELISAs) are commonly used in routine laboratory analysis and the same approach can be transferred to the LFIA platform. Quantitative information can be obtained by interpolation on pre-stored calibration curves and the reproducibility of results is increased by normalizing the T-line signal with respect to the intensity of the signal in correspondence of the C-line. Enzyme labels give the possibility of greatly amplifying the analytical signal since in the presence of an excess of substrate each enzyme molecule can produce many product molecules, which can be measured and related to the target analyte concentration.

Furthermore, depending on the enzyme/substrate system, the LFIA technique can be combined with different detection methods such as colorimetry, fluorescence, and chemiluminescence (CL). Selection of the detection principle is crucial for the dynamic ranges obtained with enzyme-based LFIAs, which are generally about two orders of magnitude and can be ad hoc optimized in relation to the analyte of interest. It is also possible to use enzymes coupled with other labels like MNPs to exploit the amplification phenomena. Lastly, artificial enzyme-mimicking systems have also been proposed in LFIA applications to overcome enzyme limitations, such as the sensitivity to the environment (e.g., temperature and pH) and the possible inhibition by interferents present in the biological sample.

This review aims to present the major recent achievements in the field of enzyme-based LFIAs showing their advantages and weaknesses as well as the possibilities for future development.

## 2. Enzyme-Labeled Conjugates in LFIA

Enzymes-labeled conjugates have limited application in LFIAs, which mainly regarded horseradish peroxidase (HRP), even though alkaline phosphatase [45,46,47,48] and cholinesterase [49] enzymes were also employed. Undoubtedly, HRP is one of the most versatile enzymes, as it can catalyze the oxidation of different organic substrates by hydrogen peroxide. Several substrates for HRP are commercially available. Chromogenic substrates—such as 3,3′,5,5′-tetramethylbenzidine (TMB), 2′-azino-bis-(3-ethylbenzothiazoline-6-sulfonic acid) (ABTS), o-phenylenediamine dihyrochloride (OPD) and diaminobenzidine (DAB)—generate colored reaction products, while CL substrates—such as luminol and lucigenin—yield photons, and FL substrates—such as p-hydroxyphenylacetic acid, 3-(p-hydroxyphenyl)-propionic acid and homovanillic acid—produce fluorescent species. One of the main reasons for the success of HRP as a label for bioanalytical applications is its high turnover number, which leads to high signal amplification factors. With respect to conventional LFIAs, the adoption of HRP-enhanced detection provided superior sensitivity, with improvements ranging from 5-fold [50] to 100-fold [51] for different antigens.

Several examples were reported based on the use of chromogenic substrates for enhancing colorimetric-based LFIAs [52,53]. Competitive LFIAs for multiplexed analysis of carbaryl and endosulfan using AuNPs and HRP as labels were developed by Zhang et al. [54] to compare the performances obtained with the different systems. In both cases the analytical signal was visually detected. For the AuNPs-based immunoassay the visual detection limits were 100 and 10 μg/L for carbaryl and endosulfan, respectively. Using HRP as label, the visual detection limits lowered to 10 μg L^−1^ for carbaryl and 1 μg L^−1^ for endosulfan, thus demonstrating a 10-fold enhancement with respect to the traditional approach.

**Figure 1:**
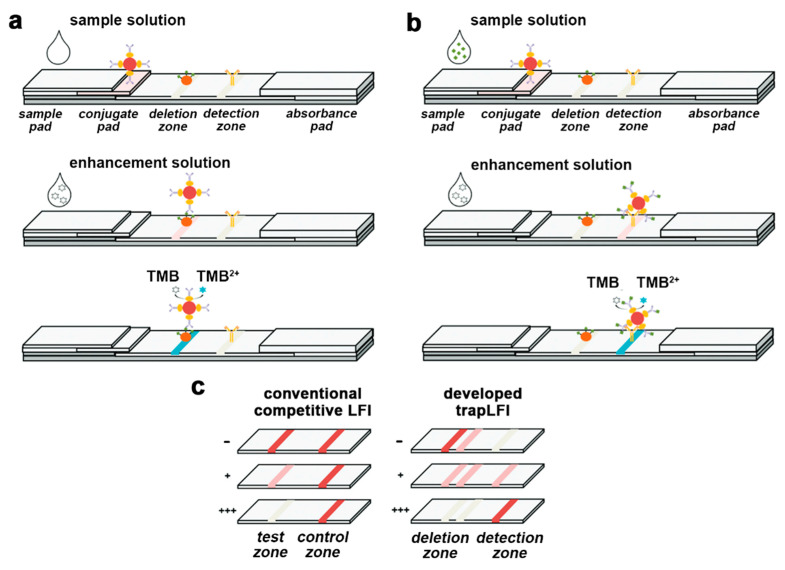
Schematic illustration of mechanism for the trapLFI biosensor with (**a**) low and (**b**) high concentration of cortisol, and (**c**) comparison of signal behavior of conventional competitive LFIA and trapLFI biosensor in the presence of different concentrations of cortisol (- negative sample, + positive sample low concentration, +++ positive sample high concentration). Adapted from Ref. [52], Copyright (2018), with permission from The Royal Society of Chemistry.

Samsonova et al., reported a LFIA method for progesterone detection in whole cow milk, in which HRP was used as a label along with a TMB substrate solution containing dextran sulfate to obtain the precipitation of a blue-colored enzyme product on the nitrocellulose membrane [55]. A low-cost paper-based platform for the early diagnosis of *Plasmodium falciparum* was developed. The assay detected the histidine-rich protein 2 of *Plasmodium falciparum* by an HRP-based colorimetric assay employing the TMB substrate. A LFIA strip was ad hoc developed using a CO_2_ laser cutter, so that the regions for sample addition, the detection zone and the absorbent pad were linked by microfluidic channels, to reduce reagents consumption [56]. An alternative design of the LFIA platform was also proposed by Oh et al., who described a “trapLFI” sensor for cortisol showing deletion and detection zones instead of the traditional T- and C-lines (Figure 1). Target-bound conjugates are captured in the detection zone, whereas conjugates not binding the targets are trapped in the deletion zone. Using this platform, the HRP color signals increase in the detection zone and decrease in the deletion zone depending on the concentration of the target analyte. The ratio of signals from deletion and detection zones allowed to quantify cortisol in a wide concentration range (0.01–100 ng mL^−1^) with high detection sensitivity (9.9 pg mL^−1^) [52].

As previously mentioned, HRP is commonly used as a label for CL detection, most often employing a luminol/H_2_O_2_/enhancer enzyme substrate [57]. CL is particularly suited for the development of portable biosensors due to its inherent sensitivity and simplicity (no specific sample geometry or excitation source are required) and because the emitted light can be measured using unexpensive sensors, such as photodiodes or compact CCD and CMOS cameras [58,59,60]. Several CL-LFIA biosensors were described in the literature [61]. Furthermore, innovative detectors such as miniaturized hydrogenated amorphous silicon photodiode arrays [62], have been used for acquiring the light signal. Such sensors represented an alternative low-cost and versatile technology for the development of portable biosensors and have been used by Zangheri et al. [63] for the quantification of serum albumin in urine. To enable widespread assay application, smartphones has been also exploited as detectors in CL-LFIA systems, allowing both acquisition of the emitted light through the embedded CMOS cameras and processing of the analytical signal to provide quantitative information about the target analyte [64].

**Figure 2:**
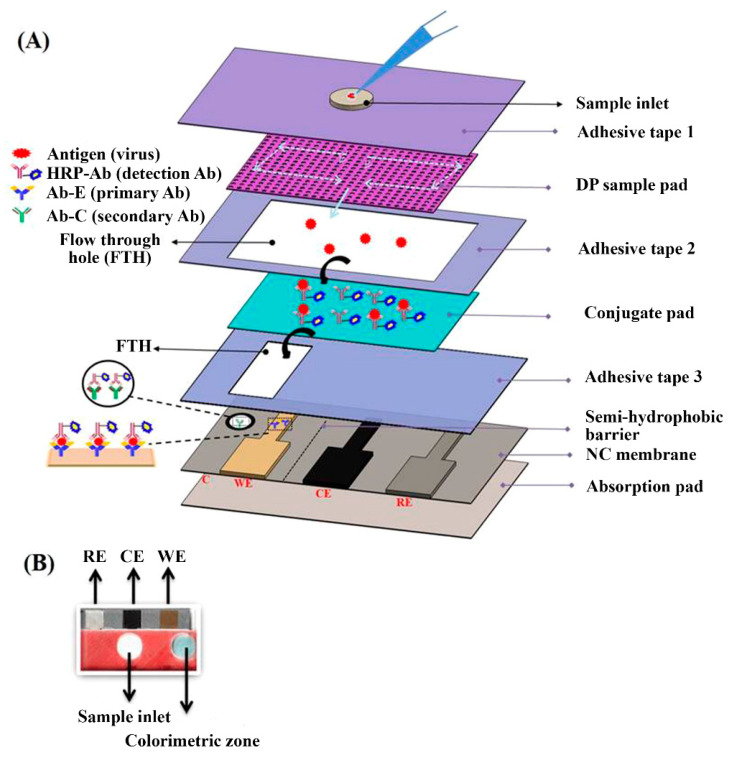
(**A**) Schematics of the integrated vertical flow assay device for dual detection. The sample pad consists of two different pore size papers: the upper one has the larger pore size (11 µm) and the lower one has the smaller pore size (0.45 µm). (**B**) Photograph of the VFA device. Adapted from Ref. [65], Copyright (2018), with permission from Elsevier.

HRP was also proposed for developing EC-LFIAs systems, by exploiting its electroactive features. As an example, a vertical-flow-based LFIA was developed for the simultaneous EC and colorimetric detection of influenza H1N1 viruses (Figure 2) [65]. In this assay, after sample injection, HRP-labeled antibodies bind the H1N1 viruses forming the (HRP-Ab)-H1N1 complexes, which are then detected on the gold electrode present on the LFIA strip through an EC-impedance-based method. This EC-LFIA system provided H1N1 viruses detection down to 3.3 and 4.7 PFU mL^−1^ in phosphate buffer saline and saliva, respectively, thus improving by 1.5-fold the performances with respect to the colorimetric approach. An enzyme-based EC- LFIA system was developed for the detection of cardiac Troponin T (cTnT) [66], in which a LFIA strip was combined with screen printed carbon electrodes. This sensor, in which cTnT detection was performed exploiting cyclic voltammetry, displayed a linear response up to 700 ng mL^−1^ of cTnT with a LOD of 0.15 ng mL^−1^.

**Figure 3:**
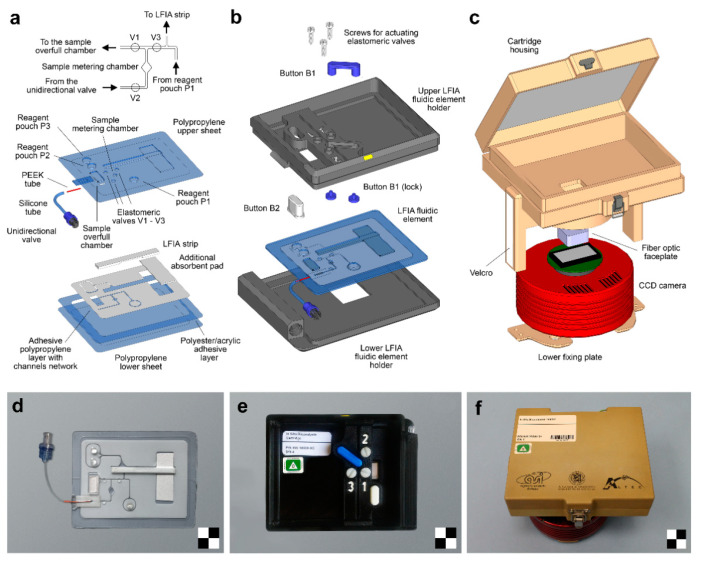
Portable CL-LFIA biosensor for the measurement of salivary cortisol levels used aboard the International Space Station (ISS): schematic drawings and images of (**a**,**d**) the LFIA fluidic element, (**b**,**e**) the LFIA cartridge and (**c**,**f**) the CL reader. A detailed view of the fluidics of the LFIA fluidic element around the sample metering chamber is shown on top of panel (**a**). Scale checkerboards are 2 × 2 cm. Reprinted from Ref. [69], Copyright (2018), with permission from Elsevier.

One of the main limitations of enzyme-based LFIA techniques is the need to add an enzyme substrate at the end of the assay for the development of the analytical signal. To accomplish this task, Cho et al., proposed a LFIA configuration consisting in two crosswise-arranged membrane pads in vertical and horizontal directions. The immunoreactions took place on the vertically arranged pads, while the horizontally arranged pads were positioned in such a way that they allowed to supply the chromogenic HRP enzyme substrate in correspondence of the T- and C-lines. Under optimal conditions, the use of HRP as label increased the assay detection capability of ~30 times compared to that of AuNPs [67]. To enable easy handling of sample and reagents, including the addition of the two-component CL substrate for HRP, microfluidic cartridges have recently been proposed for CL-LFIA. The cartridges contain the reagents required for the analysis and deliver them to the LFIA strip in the appropriate time sequence [63,68]. In this context, Zangheri et al., also proposed a portable CL-LFIA biosensor that was successfully used aboard the International Space Station (ISS) by the astronaut Paolo Nespoli (VITA mission, July–December 2017) to monitor salivary cortisol, a stress marker (Figure 3) [69]. An alternative approach relayed on a delayed release of two CL substrate components through an asymmetric polysulfone membrane placed between the nitrocellulose membrane and the substrate pad. Upon sample application, enzyme substrates encapsulated in the pad are released after 5.3 ± 0.3 min, providing an automated CL-LFIA system, which sequentially performs immunoreaction, pH change, substrate release, hydrogen peroxide generation and CL reaction simply following sample loading [70].

The use of enzyme as labels opens various detection possibilities, which can be selected according to the specific application and the available instrumentation. The combination of the increased sensitivity achievable through enzyme labels with the possibility of quantifying the analyte using simple and widespread technologies (e.g., smartphone cameras) will surely yield to significant advancements in LFIAs, which could further expand their fields of application. Efforts must be still dedicated to solving problems such as the limited shelf-life of the enzymatic reagents, the dependence of the enzymatic activity on the surrounding conditions and the implementation of multistep assay procedures. Indeed, these aspects still limit the use of such systems for routine applications.

## 3. Modification of MNPs Labels with Enzyme: Enhancing LFIA Performance

Enzymes and MNPs have been combined to further enhance the analytical signal in LFIAs. Indeed, the surface of AuNPs can be easily modified to afford secondary reactions or functionalized by suitable compounds for exploiting enzyme-based detection, most often employing HRP [14,71]. In this way, AuNPs not only acts as a direct label, but also as a carrier for multiple HRP molecules, thus obtaining further signal amplification.

**Figure 4:**
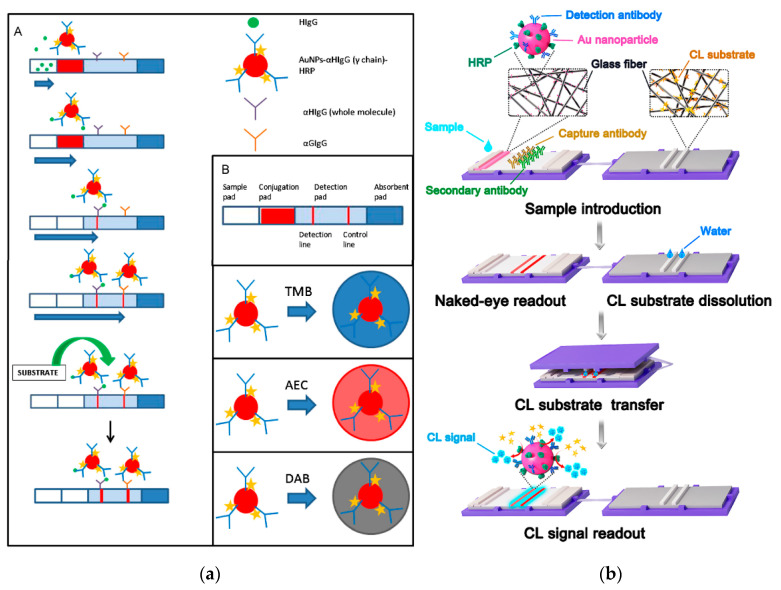
(**a**): (**A**) Scheme of the LFIA for the detection of HIgG. (**B**) Detail of the different parts of a LFIA strip and cartoons representing the AuNP modified with the antibody anti-human IgG γ chain specific HRP modified, and the different colors expected for the different substrates (TMB, AEC and DAB) used. Reprinted from Ref [72], Copyright (2012), with permission from Elsevier. (**b**) Scheme of the CLFAs for the Detection of Targets: Sample is introduced on the sample pad and moves to the absorbent pad. When sample flows through the conjugate pad, the targets are recognized by the detection antibodies on the AuNPs. With sample flowing through the NC membrane, targets and detection antibodies, as well as AuNPs, are immobilized on the NC membrane. After the appearance of the red lines because of the aggregation of AuNPs, the substrate pad containing lyophilized substrate is covered on the LFA strip for the transfer of the CL substrate. Then, the substrate pad is removed, and the CL signal is captured for quantitative detection of targets. Reprinted from Ref [76], Copyright (2018), with permission of the American Chemical Society.

Parolo et al. [72] used a LFIA system to evaluate the colorimetric substrates TMB, 3-amino-9-ethylcarbazole (AEC), and DAB for the detection of HRP molecules immobilized onto AuNPs (Figure 4a). The developed LFIAs offered two different detection alternatives, namely the direct observation of the red color produced by accumulation of AuNPs in the T- and C-lines and, to improve sensitivity, the formation of insoluble chromogens in the correspondence of the lines upon addition of the HRP chromogenic substrates. The double detection system thus provided “on-demand” tuning of the biosensing performance. Use of AEC/H_2_O_2_ as enzyme substrate provided a significant sensitivity enhancement with respect to AuNPs. Nevertheless, the single-component TMB enzyme substrate gave the lowest quantification limits thanks to the higher contrast between the lines and the background with respect to AEC. The detection limit achieved for proteinaceous target analytes was 200 pg mL^−1^.

Following the same principle, Mao et al., conjugated AuNPs with both HRP and DNA complementary probes for target DNA sequence detection [73]. Upon DNA duplex formation, target DNA was detected by addition of AEC/H_2_O_2_ substrate solution to the sample pad, which led to color product formation on the T- and C-lines, in 5 min. Thanks to the signal amplification strategy, a 10-fold improved sensitivity was obtained with respect to the traditional colorimetric AuNPs-based approach. The same research group also optimized a synthetic strategy for HRP–AuNP–DNA probe conjugate. The addition of sodium dodecyl sulfate (SDS) and the optimized sequence of immobilization of HRP and DNA probes on the AuNPs provided a further improvement of assay sensitivity [74].

CL detection has been also proposed to develop a dual-readout LFIA [75] in which the accumulation of HRP-AuNPs-labeled species in the correspondence of the T- and C-lines was detected through both the red color due to AuNPs and the CL emission produced by the HRP-catalyzed oxidation of luminol. The proposed system was used for detecting tumor biomarkers such as alpha fetoprotein (AFP) and carcinoembryonic antigen (CEA) and the bacterial infection biomarker procalcitonin (PCT) in serum samples and in whole blood.

Another work reported a self-contained CL-LFIA which prestored reagents (Figure 4b) [76]. This CL-LFIA system included the LFIA strip and the CL substrate pad in a polycarbonate holder. AuNPs simultaneously labeled with HRP and antibody were deposited on the conjugate pad while a glass fiber substrate pad contained sodium perborate as the oxidant and the lyophilized CL substrate, allowing long-term storage of the LFIA device. After transfer of the CL substrate from the substrate pad to the nitrocellulose membrane, the CL signal was acquired for the quantification of the targets. The HRP on the AuNPs efficiently amplified the CL signal and both macromolecules and small molecules could be successfully detected.

Han et al. proposed a new conjugation scheme for development of high-performance LFIAs [77]. AuNPs conjugated with aldehyde-activated HRP and antibody molecules (AuNP-(ald)HRP-Ab) were produced and employed in a highly sensitive CL-LFIA for human cardiac troponin I (cTnI), a cardiac biomarker used to diagnose myocardial infarction. The AuNP-(ald)HRP-Ab conjugate allowed a 110-fold sensitivity enhancement in comparison to a typical AuNP-Ab conjugate. This CL-LFIA allowed detection of cTnI in standards and clinical serum samples with a detection limit of 5.6 pg mL^−1^ and acceptably precision according to clinical guidelines (variation coefficient of 2.3–8.4%).

The use of MNPs conjugated to enzymes offers two distinct advantages: first, the enhancement of the analytical signal and secondly the possibility of performing a double detection. Indeed, a preliminary visual qualitative detection could allow to obtain a rapid information about the presence or absence of the target analyte, then, for positive samples, an ultrasensitive quantitative evaluation can be performed, possibly using portable detectors. Table 1 shows a comparison of the performance of various HRP-based LFIA developed for the detection of the same analyte (IgG) reported in literature [72] and in commerce [78,79].

## 4. Nanozymes

To overcome the limitations of natural enzymes (i.e., short shelf life, possible inhibition or activation due to interferents in the sample matrix, denaturation at high temperatures and acidic/alkaline pH) nanoscale materials with enzyme-like catalytic properties, defined as “nanozymes”, are attracting increasing interest [80,81,82,83].

Compared to natural enzymes, nanozymes have strong robustness against harsh environments, low cost, easy production through chemical synthesis, and tunable catalytic activity. With these advantages, they have been widely used as alternatives to natural enzymes in catalysis [84], sensing [85,86], environmental engineering [87], and biomedicine [88]. To date various nanomaterials, ranging from metals [89,90] to metal oxides [91,92], metal-organic frameworks (MOFs) [93] and carbon-based nanomaterials [94], have demonstrated to possess peroxidase-like activity.

The use of a peroxidase-like nanozyme labels as a substitute of HRP is expected to lead to nanozyme-based immunosensors exhibiting lower cost, better stability, and easier production, while retaining the original advantages of the natural enzyme, such as high sensitivity, good selectivity and high turnover [95]. Thus, the past few years have seen a growing diffusion of immunoassays and immunosensors using nanozymes as signaling elements [83] and this trend is confirmed also for LFIAs. Gao et al., showed that Au-Pt NPs retain the plasmonic activity of AuNPs while possessing increased catalytic activity due to the Pt skin. Such plasmonic and catalytic functionalities offer two different detection alternatives: one exploiting just the color deriving from AuNPs plasmonics (low-sensitivity mode) and the second relying on the nanozyme-catalyzed reaction upon addition of a chromogenic substrate (high-sensitivity mode). Using a LFIA platform it has been demonstrated that use of Au-Pt NPs as label could enhance detection sensitivity by 2 orders of magnitude with respect to conventional AuNPs [96].

**Figure 5:**
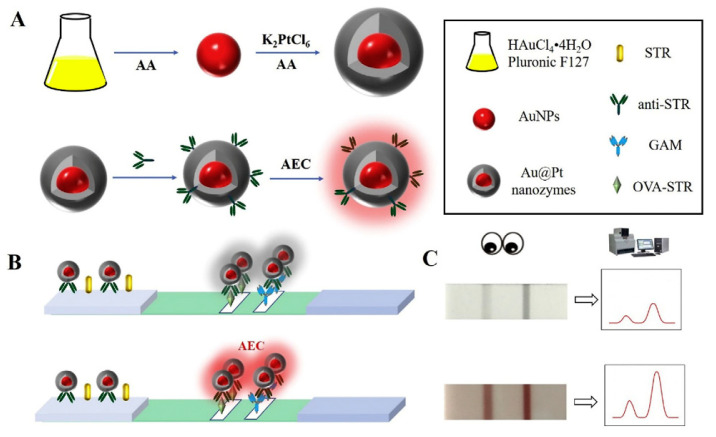
Schematic illustration for the colorimetric LFA based on Au-Pt: (**A**) preparation of Au@Pt nanozyme; (**B**) process of the colorimetric LFA based on Au-Pt; (**C**) typical results of the colorimetric LFA assay based on Au-Pt. Reprinted from Ref [97], Copyright (2020), with permission from Elsevier.

Au-Pt NPs were also recently exploited for the detection of streptomycin, a typical aminoglycoside antibiotic (Figure 5). A novel LFIA based on Au-Pt NPs as both visual tag and artificial enzyme for colorimetric detection was developed and compared with conventional LFIA based on AuNPs. The limits of detection were 1 ng mL^−1^ for the LFIA based on Au-Pt NPs as visual tag and 0.1 ng mL^−1^ for the enhanced LFIA based on the Au-Pt NPs enzyme-like activity, while a value of 8 ng mL^−1^ was obtained for the LFIA based on conventional AuNPs [97].

Porous platinum core–shell nanocatalysts (PtNCs), exhibiting high catalytic activity even when exposed to human serum samples, were conjugated to nanobodies specific for the detection of p24 antigen [98]. The optimal nanoparticle size was established, ensuring efficient amplification and performance in LFIA, thus enabling naked-eye detection of p24 spiked into sera down to 0.8 pg mL^−1^ and diagnosis of acute-phase HIV in clinical human plasma samples in less than 20 min.

Jiang et al. [99] developed a LFIA method for *E. coli* O157:H7 based on the use of bimetallic Pt–Au NPs as colorimetric label. The signal amplification is based on the high peroxidase activity of Pt–Au NPs which, upon addition of TMB, can produce a characteristic-colored precipitate, thus enabling visual detection without instrumentation. The innovative aspect of this approach lies in the quantification of the target pathogen through the measurement of color intensity. Due to the excellent peroxidase activity of Pt–Au NPs, a strong color, clearly visible at the naked eye, was obtained in less than 1 min even in the presence of low amounts of *E. coli* O157:H7. Quantification was performed using a commercial assay meter. A more than 1000-fold sensitivity improvement was obtained compared to the conventional LFIA based on AuNPs.

**Figure 6:**
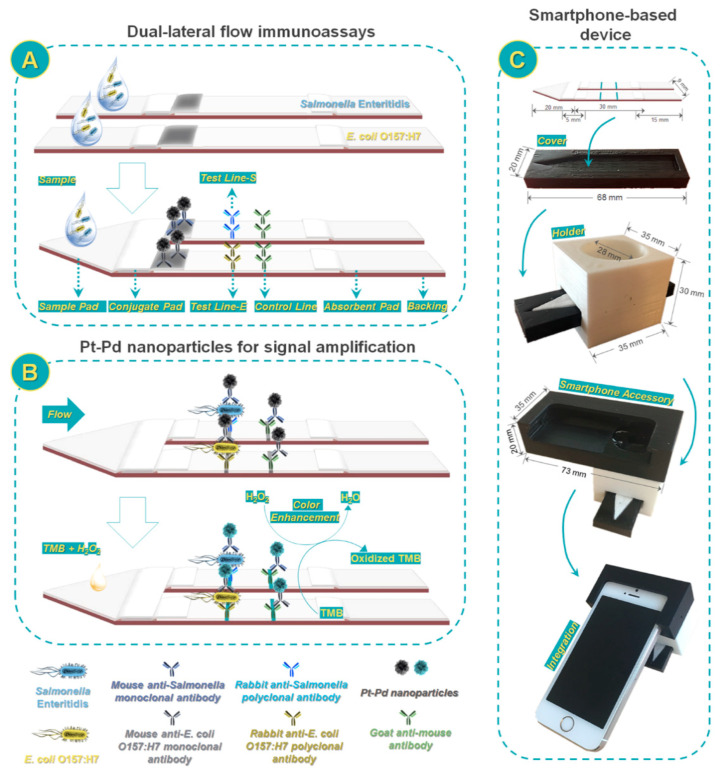
Scheme of smartphone-based dual lateral flow immunoassays for simultaneous detection of *S. enteritidis* and *E. coli* O157:H7 using Pd@Pt nanoparticles as signal amplification: (**A**) Dual lateral flow immunoassays; (**B**) Pd@Pt nanoparticles for signal amplification; and (**C**) smartphone-based device. Reprinted from Ref. [101], Copyright (2017), with permission from the American Chemical Society.

Another dual detection method for the rapid detection of *E. coli* O157:H7 was developed exploiting a nanozyme-bacteria-antibody sandwich pattern. A type of functional nanozyme—mannose modified Prussian blue (man-PB) was introduced which acted both as the recognition agent and as tracer. Apart from original signal intensity on the T-line, the peroxidase-like catalytic activity-driven generation of colorimetric signal could be used as an alternative for quantification purposes [100]. In the context of bacteria detection, Cheng et al. [101] developed a multiplex LFIA for simultaneous quantification of *Salmonella enteritidis* and *E. coli* O157:H7 based on the use of antibody-modified Pt-Pd NPs. After target recognition, the peroxidase-mimicking activity of the Pt-Pd NPs was exploited to obtain a blue color in the presence of TMB and H_2_O_2_. To enable on site application, a portable smartphone-based device was fabricated to record and analyze the colorimetric results (Figure 6). After assay optimization, LODs of about 20 and 34 CFU mL^−1^ were obtained for *Salmonella enteritidis* and *E. coli* O157:H7, respectively.

A LFIA based on PB nanozyme was also proposed by Liu et al. [102]. The authors reported about a magnetic-PB nanozyme which enables a colorimetric simultaneous detection of ractopamine (RAC) and clenbuterol (CLE). They exploited both a visual and a quantitative approach obtaining a dual-readout strategy and they evaluated the improvements in the performances of the method by the exploitation of the catalytic properties of the nanozyme label. Indeed, the LOD obtained by visual approach was 1.0 ng mL^−1^ both for RAC and CLE, while it decreased to 0.12 and 0.20 ng mL^−1^ for RAC and CLE, respectively by measuring the colorimetric intensity of the lines.

Another work reported the preparation of gold-platinum nanoflowers (AuPt NFs) and their simultaneous use as colorimetric and enzyme mimicking label in LFIA. The AuPt NFs were prepared by growing Pt nanowires on the surface of gold nanoparticles. The assay involved the capture of the target (rabbit IgG was used as a model analyte) by an immobilized capture antibody and the formation of a sandwich with an AuPt NF-labeled second antibody. The immunosandwich is captured by the T-line producing a characteristic black band enabling visual detection of the target IgG. The coloration of the T-line can be enhanced by addition of the chromogenic substrate AEC, which is catalytically oxidized by the Pt nanowires on the AuPt NFs giving a red color. Finally, a portable strip reader was used to measure the test line intensities and calculate the IgG concentration. The LFIA has a 5 pg mL^−1^ detection limit for IgG under optimized experimental conditions, which is 100 times lower than that obtained by the conventional AuNP-based LFIA [103].

Manganese dioxide nanoflowers (MnO_2_-NFs) were also proposed [104] as a dual readout probe in a LFIA for detecting chlorpyrifos residues. The MnO_2_-NF label led to a brown color on the T-line, which could be easily observed by the naked eye to provide a qualitative readout. Due to the small colorimetric differences observed with trace levels of chlorpyrifos, the readout by naked eyes could not meet the demand of quantitative analysis. Nevertheless, as MnO_2_-NFs showed a significant catalytic effect on the luminol/H_2_O_2_ CL system, the CL signal was used to quantitatively detect chlorpyrifos at trace levels. Quenching studies using 1,3-diphenylisobenzofuran and colorimetric assays with TMB/H_2_O_2_ as chromogenic substrate were conducted to unravel the enhancing mechanism of MnO_2_-NFs, in which the oxidant activity of MnO_2_ caused the decomposition of H_2_O_2_ leading to reactive oxygen species. Under optimal conditions, the linear range of detection of chlorpyrifos was 0.1–50 ng mL^−1^ with a detection limit of 0.033 ng mL^−1^.

Another approach was reported by Bu et al. [105], based on the use of molybdenum disulfide (MoS_2_) nanosheets as label for sensitive LFIA applied to the detection of tetracycline. Under optimized experimental conditions, the developed biosensor showed a visual LOD of 0.023 ng mL^−1^, while exploiting a portable colorimetric LFIA strip reader it was possible to achieve a LOD of 0.012 ng mL^−1^.

Nanozymes might represent the turning point for the development of enzyme enhanced LFIA systems, as they combine the advantages of enzyme labels with the stability of nanomaterials. Anyhow, when comparing the pros and cons in substituting enzymes with nanozymes, it is important to consider the costs of the nanozymes synthesis. Indeed, one of the reasons of the LFIA’s success is the use of very inexpensive materials. Even when AuNPs labels are used, the limited amount of material required for the preparation of a strip and the easy synthetic procedure make LFIAs very affordable. On the contrary, nanozymes are usually synthesized by employing complex and expensive procedures. Therefore, their wide applicability depends on the improvement of procedures for the large-scale production of nanozymes. Furthermore, a still open issue is the delivery of the enzyme substrate, which requires an additional step in comparison to conventional visual LFIA systems. Anyhow, this problem can be solved with ad hoc solutions such as the deposition of the reagents in dry form on the strip or the use of automated system or preloaded cartridges for dispensing the enzyme substrate during the detection step.

## 5. Conclusions

Enzymatic labels have found widespread use in routine immunoassays and are widely applied in clinical laboratories. Their transfer to LFIA platforms is potentially a great step forward, as it combines the advantages of the ELISA methods (high sensitivity, selectivity and specificity) with those of the LFIA technique (rapidity, portability and ease-of-use). The use of enzymes alone or coupled with MNPs as labels for LFIAs has already led to significant advances in terms of sensitivity and of range of applicability. Some of the problems related to the on-site use of enzyme labels, such as susceptibility to ambient conditions, limited shelf life and specific requirements for storage, can be overcome by nanozymes. The works reported in this Review are summarized in Table 2. 

However, each of these approaches has advantages and disadvantages, as briefly listed in Table 3. Moreover, there are still some open issues, for example about the storage of enzyme based LFIA systems and the delivering of the enzyme substrate, which requires an additional procedural step and often a dedicated microfluidics.

The future of this technique is therefore linked to a combined approach involving the study of new nanomaterials for the storage and release of the reagents, the synthesis of innovative enzymatic tracers and the design of new microfluidic pathways that allow to activate the reagents at the proper time. Whichever will be the final technical solution, the impact of enzyme-based lateral flow technology will be greatly enhanced by innovative solutions and we can expect to see considerable innovation in this area in the coming years.

## Figures and Tables

**Table 1:** Comparison of performance of HRP-based LFIAs for the detection of IgG (for reference, conventional AuNP-based LFIAs and ELISAs for IgG are also reported as the first and last table entries, respectively).

Technique	Detection Principle	Tracer	Limit of Detection	Reference
Colorimetric LFIA	Visual detection	AuNP	50 ng mL^−1^	[72]
Colorimetric LFIA	Measurement of color intensity with a portable strip reader	AuNP	2 ng mL^−1^	[72]
Colorimetric LFIA	Visual detection	HRP with metal enhancer (AuNPs) using TMB substrate	5 ng mL^−1^	[72]
Colorimetric LFIA	Measurement of color intensity with a portable strip reader	HRP with metal enhancer (AuNPs) using TMB substrate	200 pg mL^−1^	[72]
Colorimetric LFIA	Visual detection	HRP with metal enhancer (AuNPs) using AEC substrate	5 ng mL^−1^	[72]
Colorimetric LFIA	Measurement of color intensity with a portable strip reader	HRP with metal enhancer (AuNPs) using AEC substrate	310 pg mL^−1^	[72]
Colorimetric LFIA	Visual detection	HRP with metal enhancer (AuNPs) using DAB substrate	50 ng mL^−1^	[72]
Colorimetric LFIA	Measurement of color intensity with a portable strip reader	HRP with metal enhancer (AuNPs) using DAB substrate	1.6 ng mL^−1^	[72]
Colorimetric ELISA	Measurements of absorbance using a microwell plate reader	HRP using TMB substrate	0.24 ng mL^−1^	[78]
Colorimetric ELISA	Measurements of absorbance using a microwell plate reader	HRP using TMB substrate	20 pg mL^−1^	[79]

**Table 2:** Scheme of the work reported in literature exploiting enzyme-based LFIA.

Technique	Detection Principle	Analyte	Limit of Detection	Reference
Enzyme as tracer	HRP (colorimetric)	Carbaryl, endosulfan	10 μg L^−1^ (carbaryl), 1 μg L^−1^ (endosulfan)	[54]
	HRP (colorimetric)	Progesterone	0.8 ng mL^−1^	[55]
	HRP (colorimetric)	Histidine-rich protein 2 of *Plasmodium falciparum*	4.5 ng mL^−1^ (59 parasites μL^−1^)	[56]
	HRP (colorimetric)	Cortisol	9.9 pg mL^−1^	[52]
	HRP (chemiluminescent)	Human serum albumin (has)	2.5 mg L^−1^	[63]
	HRP (chemiluminescent)	Cortisol	0.3 ng mL^−1^	[64]
	HRP (chemiluminescent)	Hepatitis B surface antigen	0.12 ng mL^−1^	[67]
	HRP (chemiluminescent)	Cortisol	0.2 ng mL^−1^	[69]
	HRP (chemiluminescent)	C-reactive protein	1.05 ng mL^−1^	[70]
	HRP (electrochemical)	H1N1 viruses	4.7 PFU mL^−1^	[65]
	HRP (electrochemical)	Troponin T	0.15 ng mL^−1^	[66]
Modification of MNPs labels with enzyme	AuNP-HRP (colorimetric)	IgG	200 pg mL^−1^	[72]
	AuNP-HRP (colorimetric)	DNA sequence	0.01 pM	[74]
	AuNP-HRP (chemiluminescent)	α-fetoprotein (AFP), carcinoembryonic antigen (CEA), procalcitonin (PCT)	0.2 ng mL^−1^ (CEA), 0.21 ng mL^−1^ (AFP), 0.02 pg mL^−1^ (PCT)	[75]
	AuNP-HRP (chemiluminescent)	α-fetoprotein (AFP), folic acid (FA)	0.27 ng mL^−1^ (AFP), 0.1 ng mL^−1^ (FA)	[76]
	AuNP-HRP (chemiluminescent)	Human cardiac Troponin I	5.6 pg mL^−1^	[77]
Nanozyme	Au-Pt NPs (colorimetric)	Human prostate-specific antigen (PSA)	3.1 pg mL^−1^	[96]
	Au-Pt NPs (colorimetric)	Streptomycin	0.1 ng mL^−1^	[97]
	Porous platinum core–shell nanocatalysts (PtNCs) (colorimetric)	p24 antigen	0.8 pg mL^−1^	[98]
	Pt–Au NPs (colorimetric)	*E. coli* O157:H7	10^2^ cells mL^−1^	[99]
	Mannose modified Prussian blue (colorimetric)	*E. coli* O157:H7	10^2^ cfu mL^−1^	[100]
	Pt-Pd NPs (colorimetric)	*Salmonella enteritidis*, *E. coli* O157:H7	20 CFU mL^−1^ (*Salmonella enteritidis*), 34 CFU mL^−1^ (*E. coli* O157:H7)	[101]
	Magnetic Prussian blue (colorimetric)	Ractopamine (RAC) and Clenbuterol (CLE)	0.12 ng mL^−1^ (RAC), 0.20 ng mL^−1^ (CLE)	[102]
	Gold-platinum nanoflowers (AuPt NFs) (colorimetric)	IgG	5 pg mL^−1^	[103]
	Manganese dioxide nanoflowers (MnO_2_-NFs) (colorimetric)	Chlorpyrifos	0.033 ng mL^−1^	[104]
	Molybdenum disulfide (MoS_2_) nanosheets (colorimetric)	Tetracycline	0.012 ng mL^−1^	[105]

**Table 3:** Scheme of the enzymatic-based tracers used in LFIA compared with the traditional MNPs labels.

Tracer	Detection	Pros	Cons
Metal Nanoparticles (MNPs)	Qualitative information by visual detection; Quantitative information by measuring the intensity of the color using portable strip reader	No need to add reagents and substrate; MNPs can be immobilized on the conjugate pad and easily resolubilized by dispensing the sample; Stability; Long shelf-life	Relatively high detection limit
Enzyme	Quantitative information can be obtained exploiting colorimetric, chemiluminescent, or electrochemical approach; Exploiting colorimetric approach, it is possible to obtain a qualitative response by visual detection	Higher sensitivity with respect to the use of MNPs; Lower limit of detection (from 5-fold to 100-fold with respect to the use of MNPs)	Addition of substrate; Limited shelf-life; The enzyme is better preserved in solution rather than adsorbed on paper-based materials
Enzyme with MNPs	Qualitative visual information due to the accumulation of MNPs on the lines; Quantitative information obtained by adding the proper enzymatic substrate (colorimetric or chemiluminescent detection)	The surface of MNPs can be easily modified to afford secondary reactions or functionalized by suitable compounds for exploiting enzyme-based detection; AuNPs not only act as a direct label, but also as a carrier for multiple enzyme molecules, thus obtaining further signal amplification	Addition of substrate Limited shelf-life
Nanozyme	Qualitative visual information due to the accumulation of tracers on the lines; Quantitative information obtained by adding the proper enzymatic substrate (colorimetric or chemiluminescent detection)	Higher stability; Longer shelf-life; Easily immobilized on paper-based materials	Addition of enzymatic substrate High cost
